# Oropouche Virus: An Emerging Orthobunyavirus

**DOI:** 10.1099/jgv.0.002027

**Published:** 2024-10-01

**Authors:** Natasha L. Tilston-Lunel

**Affiliations:** 1 Department of Microbiology and Immunology, Indiana University School of Medicine, Indianapolis, Indiana, USA

**Keywords:** arbovirus, emerging virus, Oropouche virus, orthobunyavirus, reassortment

## Abstract

On 2 February 2024, the Pan American Health Organization/World Health Organization issued an epidemiological alert on rising Oropouche virus (OROV) infections in South America. By 3 August 2024, this alert level had escalated from medium to high. OROV has been a public health concern in Central and South America since its emergence in Brazil in the 1960s. However, the 2024 outbreak marks a turning point, with the sustained transmission in non-endemic regions of Brazil, local transmission in Cuba, two fatalities and several cases of vertical transmission. As of the end of August 2024, 9852 OROV cases have been confirmed. The 2024 OROV outbreak underscores critical gaps in our understanding of OROV pathogenesis and highlights the urgent need for antivirals and vaccines. This review aims to provide a concise overview of OROV, a neglected orthobunyavirus.

## Perspective

Oropouche virus (OROV, *Orthobunyavirus oropoucheense*) [1], an obscure arbovirus that emerged in the mid-1950s to become a significant public health concern in Central and South America, is now gaining global attention. Between January and August 2024, 9852 confirmed OROV cases have been reported, including five cases of vertical transmission, four possible congenital malformations, and two deaths in young adults with no comorbidities. These are the first documented cases of vertical transmission and mortalities due to OROV [2,3]. The World Health Organization (WHO) has assessed the overall public health risk posed by OROV as high at both regional and global levels [4]. The rising incidence and severity of this OROV outbreak is a clear wake-up call, underscoring the urgent need to prepare for an anticipated increase in zoonotic events. This review presents a comprehensive overview of OROV and highlights key unanswered questions.

## OROV emergence and the current outbreak

**Fig. 1.**
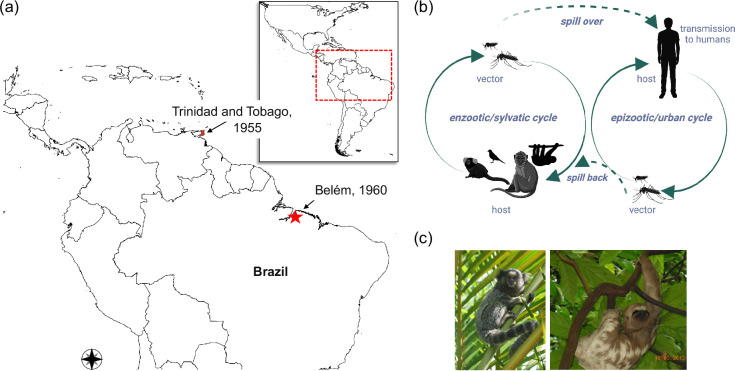
OROV emergence and transmission cycle. (**a**) Representation of the approximate geographic location of OROV emergence in Trinidad and Brazil. The map was generated using QGIS 3.38 [97]. (**b**) OROV transmission cycles. The sylvatic cycle involves various susceptible mammals and arthropods, and the urban cycle involves humans and primarily the biting midge *Culicoides parenesis*. The solid lines represent the independent sylvatic and urban cycles, while the dotted lines show their connection. OROV can ‘spill over’ into humans to initiate the urban cycle and ‘spill back’ into mammals and their vectors, thus maintaining OROV’s transmission cycle. The figure was created with Biorender.com (**c**) Images of potential OROV reservoirs, a marmoset and a sloth. *Personal images taken in Brazil, 2013*.

OROV first emerged in 1955 when a forest worker in Trinidad fell ill. Five years later, in 1960, OROV emerged again, this time in northern Brazil (Belém, Pará) in a pale-throated three-toed sloth [5,7] (Fig. 1a). The initial Trinidadian case involved a 24-year-old male from the village of Vega de Oropouche, from which the virus derives its name. Although no additional febrile cases were reported, serological surveys detected neutralizing antibodies against OROV in 3 out of 46 people tested and in the local primate population, indicating prior exposure [5]. However, OROV’s emergence in Brazil sparked the first-ever recorded outbreak, with an estimated 11 000 cases in 1961 in Belém, Pará. Between 1978 and 1981, approximately 220000 cases were reported in Pará, Amazonas and Amapá states. By 1988, the virus spread to Maranhão and Goiás, resulting in about 200 reported illnesses. OROV outbreaks soon emerged in different regions along the Amazon River, with more than 30 outbreaks recorded between 1961 and 1996, totalling an estimated 500 000 cases [5,13]. Recurring OROV outbreaks have continued to be reported since then in Amazonas (2007–2008), Amapá (2008–2009) and Pará (2003–2004 and 2006), as well as isolated cases popping up in the non-endemic parts of Brazil (2004–2016) [14,19]. Beyond Brazil, OROV was first reported in Panama in 1989 [20], Peru in 1992 [20], Ecuador in 2004 [21], Bolivia in the mid-2000s [22], Venezuela in 2007 [20], Haiti in 2014 [23], Columbia in 2017 [24], French Guiana in 2021 [25] and now Cuba in 2024 [26]. The true impact of OROV has been grossly underestimated. The clinical manifestations of OROV closely resemble those of other arboviral infections, such as dengue. Consequently, many OROV cases have only been identified through retrospective studies [10,27, 28]. Recorded OROV outbreaks found in the literature are listed in Table 1*.*

**Table 1.** Recorded OROV outbreaks/cases, 1954–2021

County	State	Location	Year	Month	Estimated cases	Reference
Trinidad	Sangre Grande	Vega de Oropouche	1954	September	–	[5]
Brazil	Para	Belém	1961	February–May	11 000	[7]
Brazil	Para	Caratateua	1967	February–March	400	[7]
Brazil	Para	Braganca	1967	March–July	6000	[7]
Brazil	Para	Belém	1968	February–July	n/a	[7]
Brazil	Para	Baiao	1972	June–September	85	[7]
Brazil	Para	Santarem	1974	n/a	n/a	[98]
Brazil	Para	Itupiranga	1975	May–June	420	[7]
Brazil	Para	Santarem	1975	February–April	14 000	[99]
Brazil	Para	Alter do Chao	1975	July–August	280	[7]
Brazil	Para	Mojui dos Campos	1975	December–April	600	[7]
Brazil	Para	Palhal	1975	February–April	420	[7]
Brazil	Para	Belterra	1975	April–June	1600	[7]
Brazil	Para	Tome-Aci	1978	June–October	2000	[7]
Brazil	Para	Belém	1979	April–June	16 000	[10]
Brazil	Para	Various	1979	March–November	9000	[10]
Brazil	Para	Belém	1980	February–Oct	102000	[10]
Brazil	Para	Various	1980	March–August	37 000	[10]
Brazil	Amapá	Mazagão	1980	n/a	n/a	[10]
Brazil	Amazonas	Barcelos	1980	May–June	171	[10]
Brazil	Amazonas	Manaus	1980–1981	November–March	97000	[10]
Brazil	Amapá	Mazagão	1981	n/a	n/a	[31]
Brazil	Maranhão	Porto Franco	1987–1988	December–March	130	[10,100]
Brazil	Goiás	Tocantinopolis	1988–89	December–March	n/a	[10,100, 101]
Panama	Panama	Chame/San Miguelito	1989	September	n/a	[10]
Panama	Panama Oeste	Bejuco	1989	September	n/a	[10,98]
Panama	Panama	Chilibre	1990	n/a	n/a	[10,98]
Panama	Panama	San Miguelito	1990	n/a	n/a	[10,98]
Brazil	Rondonia	Ariquemes	1991	February–March	58 574	[10]
Brazil	Rondonia	Quro Preto do Oeste	1991	February–March	35 413	[10]
Peru	Loreto	Iquitos	1992	January–April	n/a	[10]
Brazil	Pará	Serra Pelada	1994	November–December	5000	[12,100]
Peru	–	Madre de Dios	1994	n/a	120	[102]
Peru	Madre de Dios	Puerto Maldonado	1994	n/a	n/a	[13,102, 103]
Brazil	Pará	Oriximina	1996	April–May	n/a	[100]
Brazil	Pará	Altamira	1996	February–June	n/a	[31]
Brazil	Pará	Brasil Novo	1996	January–February	n/a	[100]
Brazil	Amazonas	Novo Airao	1996	March–May	n/a	[100]
Brazil	Pará	Vitoria do Xingu	1996	n/a	n/a	[100]
Brazil	Acre	Xapuri	1996	March–April	n/a	[31,100]
Peru	Loreto	Iquitos	1996–1997	n/a	n/a	[13]
Peru	Loreto	Iquitos	1998	n/a	n/a	[13,22, 102]
Peru	Loreto	Santa Clara	1998	n/a	n/a	[13,22, 102, 104]
Peru	Madre de Dios	Puerto Maldonado	1998	n/a	n/a	[13,22, 102]
Peru	Loreto	Iquitos	2000	n/a	n/a	[13,22, 102]
Peru	Madre de Dios	Puerto Maldonado	2000	n/a	n/a	[13,22, 102, 103]
Ecuador	Pastaza	n/a	2001	n/a	n/a	[105]
Brazil	Pará	Paraua-pebas	2003	April–May	n/a	[14]
Ecuador	Guayas	n/a	2003	n/a	n/a	[22]
Brazil	Acre	n/a	2004–2006	n/a	n/a	[15]
Brazil	Pará	Porto de Moz	2004	July–August	n/a	[14]
Brazil	Acre	Acrelândia	2004	July–August	n/a	[14]
Ecuador	Pastaza	n/a	2004	n/a	n/a	[105]
Argentina	Jujuy	n/a	2005	n/a	n/a	[98]
Bolivia	Cochabamba	n/a	2005–2007	n/a	n/a	[22]
Brazil	Pará	Braganca	2006	April–August	18 000	[16]
Venezuela	n/a	n/a	2007	n/a	n/a	[20]
Ecuador	Guayas	n/a	2007	n/a	n/a	[22]
Peru	Cusco	n/a	2007	n/a	n/a	[22]
Peru	Loreto	Iquitos	2007	n/a	n/a	[13,22, 102]
Peru	Madre de Dios	Puerto Maldonado	2007	n/a	n/a	[13,22, 102]
Brazil	Amazonas	Manaus	2007–2008	November–March	128	[106]
Brazil	Amapá	Mazagão	2009	June–October	n/a	Personal communication, IEC
Peru	San Martín	Bagaz’an	2010	May	108	[107]
Peru	Cajamarca	Casa Blanca	2011	n/a	n/a	[108]
Haiti	–	Gressier	2014	n/a	n/a	[23]
Ecuador	–	Esmeraldas	2016	n/a	n/a	[21]
Peru	–	Cusco	2016	January–March	57	[102]
Peru	Cusco	Echarate	2016	n/a	n/a	[109]
Peru	Cusco	Kimbiri	2016	n/a	n/a	[109]
Peru	Cusco	Lares	2016	n/a	n/a	[109]
Peru	Cusco	Palma Real	2016	n/a	n/a	[109]
Peru	Cusco	Pichari	2016	n/a	n/a	[109]
Peru	Cusco	Quebrada Honda	2016	n/a	n/a	[109]
Peru	Cusco	Quellouno	2016	n/a	n/a	[109]
Peru	Cusco	Yuveni	2016	n/a	n/a	[109]
Peru	Madre de Dios	Puerto Maldonado	2016	n/a	n/a	[13,22, 102]
Columbia	–	Turbaco	2017	September	n/a	[24]
French Guiana	–	Saul	2021	August–September	28	[25]

IECInstituto Evandro Chagasn/anot available

The emergence of OROV is a classic example of how anthropogenic changes drive zoonotic transmission. Between the 1950s and 1980s, Brazil underwent significant ecological disruption. The construction of the Belém–Brasília highway between 1958 and 1960 alone resulted in a substantial loss of the Amazon rainforest [29,30]. The sloth from which OROV was first isolated in Brazil was found near this construction site. Similarly, the 1955 Trinidad case occurred in a heavily deforested area near the Melajo forest [5]. These major deforestation events coincided with the identification of nearly 200 arboviruses in Brazil [31], making the emergence of OROV unsurprising. OROV likely originated from a sylvatic cycle involving susceptible mammals, such as non-human primates (e.g. *Callithrix penicillate* and *Cebus olivaceus*) and pale-throated three-toed sloths (*Bradypus tridactylus*) with arthropod vectors such as mosquitoes and midges [5,32,34]. While the primary transmission vector in OROV’s sylvatic cycles remains unknown, the virus was isolated from the mosquito species *Mansonia venezuelensis* in Trinidad and *Aedes serratus* in Brazil during its emergence in the 1960s[5,7, 35]. Since then, OROV RNA has also been found in other field-caught mosquitoes such as *Aedes scapularis*, *A. serratus*, *Culex fatigans* and *Psorophora ferox* [5]. When OROV spills over into humans, it can initiate an urban cycle where the likely vector is the biting midge *Culicoides paraensis* [35,36] (Fig. 1b and c). OROV-neutralizing antibodies have also been detected in wild and domestic birds [6], raising speculation about their role as potential carriers capable of spreading OROV.

**Fig. 2.**
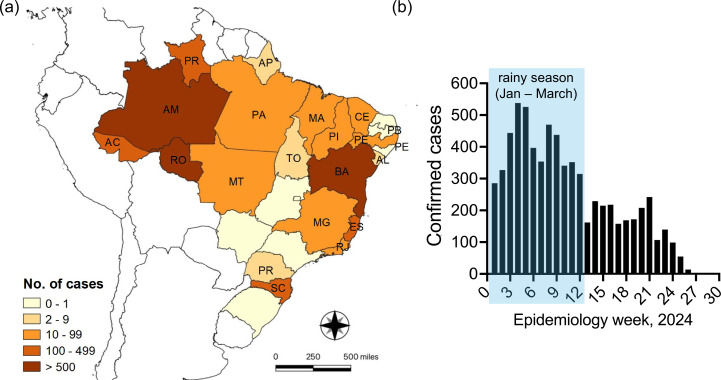
The 2024 OROV outbreak in Brazil. (**a**) Map of Brazil showing the cumulative cases by state from January 2024 to July 2024. AC, Acre; AP, Amapá; AL, Alagoas; AM, Amazonas; BA, Bahia; CE, Ceará; ES, Espírito Santo; PA, Pará; PB, Paraiba; PE, Pernambuco; PI, Piauí; PR, Pará; PR, Parana; MA, Maranhão; MG, Minas Gerais; MT, Mato Grosso; RO, Rondonia; RJ, Rio de Janeiro; SC, Santa Catarina; TO, Tocantins. (**b**) Number of confirmed OROV-positive cases by epidemiologic week covering January 2024 to July 2024. Data for the map and graph were collected from the Pan American Health Organization (PAHO)/WHO epidemiological alerts and updates [2,3, 19, 39,43, 58]. The map was generated using QGIS 3.38 [97], and the graph was plotted in GraphPad Prism 10.

**Fig. 3.**
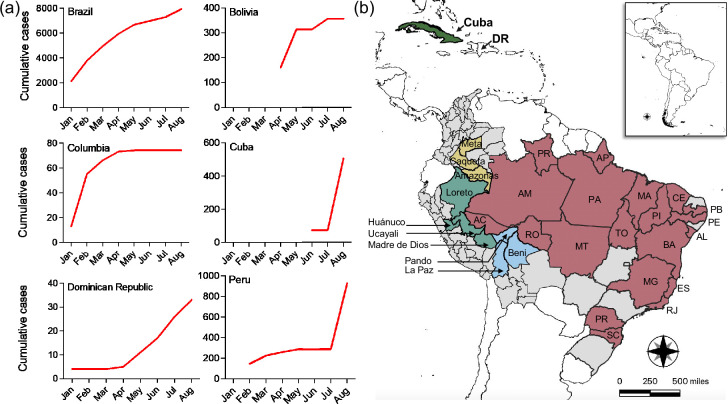
The 2024 OROV outbreak. (**a**) Cumulative OROV cases in Brazil, Bolivia, Columbia, Cuba, Dominican Republic and Peru. (**b**) OROV-affected areas highlighted: Brazil (pink), Peru (cyan), Bolivia (blue) and Columbia (sandy gold). Cuba is highlighted in green, while the Dominican Republic (DR) is left uncolored. Data for the graphs and map were sourced from PAHO/WHO epidemiological alerts and updates (last update 6 September 2024). The data may differ from actual numbers due to limitations in the available information [2,3, 19, 39,43, 58]. The map was generated using QGIS 3.38 [97], and the graphs were plotted in GraphPad Prism 10.

Historically, OROV outbreaks have been concentrated in northern Brazil. However, the 2024 outbreak marks the first time that non-endemic regions, including Bahia, Ceará, Pernambuco, Piauí, Espírito Santo, Minas Gerais, Rio de Janeiro, Mato Grosso, Mato Grosso do Sul and Santa Catarina, have experienced sustained local transmission [37,38]. The state of Amazonas in northern Brazil is likely the epicentre, with the north accounting for over 80% of all OROV cases, which is a staggering 200-fold increase over the past decade [37,38] (Fig. 2a). Based on molecular epidemiological data, Naveca *et al*. [18] reckon the first wave of the current outbreak likely began in late 2022 to early 2023 and was followed by a more significant second wave starting in October 2023 and peaking during the Amazon region’s rainy season (Fig. 2b). OROV outbreaks typically coincide with the rainy season, likely due to increased vector density. However, the authors point out that the current outbreak has unusually spanned two rainy seasons [18]. Sustained local transmission of OROV has also been confirmed in Bolivia, Columbia, Dominican Republic, Peru and, for the first time, Cuba [2,3, 19, 39,43] (Fig. 3). Poor surveillance, lack of differential diagnosis and political instability possibly hinder the complete assessment of this outbreak across other parts of South America.

**Fig. 4.**
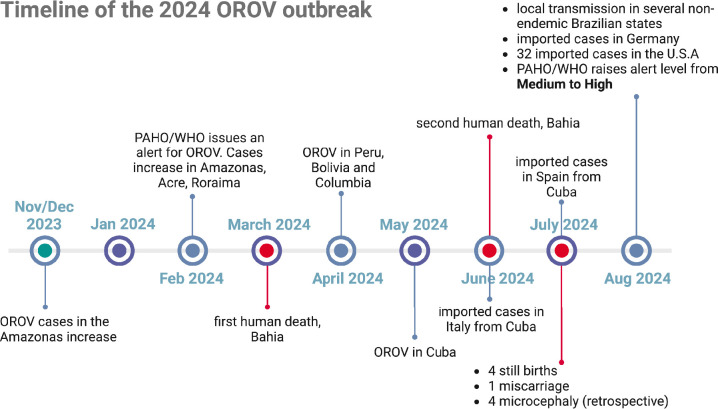
Timeline of the 2024 OROV outbreak. Months of notable occurrences are shown. Circles in red highlight the months in which mortalities were reported. Data were collected from the PAHO/WHO and the European Centre for Disease Prevention and Control’s epidemiological updates [2,3, 19, 26, 39,45, 58]. The figure was created with Biorender.com.

For the first time, Europe and the United States have reported imported OROV cases [26,44,46]. Between June and July 2024, 19 OROV-positive travellers (18 from Cuba and one from Brazil) were documented in Italy, Spain and Germany. By August 2024, 32 OROV-positive US travellers returning from Cuba had been recorded in Florida (31 cases) and New York (one case) [46,47] (Fig. 4). While the immediate risk of local transmission in Europe and the USA remains low, it is concerning. Investigations are underway to determine if local US mosquitoes (*Culex* [48] and *Aedes* [49] spp.) and midges (*Culicoides* spp. [50]) can transmit OROV. These studies are critical in assessing whether imported cases could trigger local transmission within the USA [46].

## Oropouche fever

**Fig. 5.**
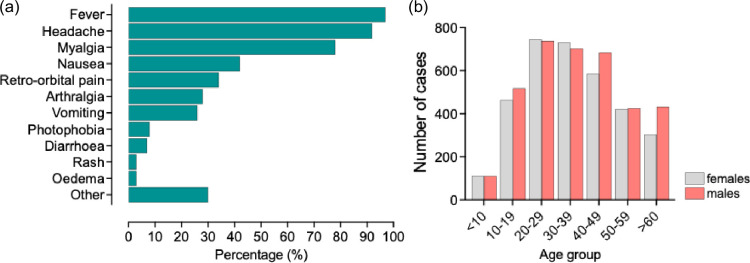
OROV symptoms. (**a**) Typical OROV symptoms. Signs and Symptoms recorded from patients in Amazonas in 2024. (**b**) Number of OROV cases classified by age and gender. Data for graphs was collected from [2,3, 19, 39,43, 58, 110] and plotted in GraphPad Prism 10.

Human OROV disease, or Oropouche fever, typically begins with a fever ranging from 100.4 to 104 °F, followed by headache, chills, malaise, myalgia and arthralgia [51] (Fig. 5a). The incubation period is 3 to 10 days, with viraemia peaking on day 2 after symptom onset. Severe symptoms can include vomiting and bleeding, such as petechiae, epistaxis and gingival bleeding. Lymphopaenia, leucopenia, elevated liver enzymes and thrombocytopaenia are common. The acute phase usually lasts 2 to 7 days, but in cases involving the central nervous system (CNS), it can extend up to 2–4 weeks, potentially leading to meningitis or encephalitis [52,55]. Neurological signs include severe headache, dizziness, vertigo, lethargy, nystagmus and neck stiffness. In cases with CNS involvement, viral RNA (vRNA) has been detectable in the cerebrospinal fluid (CSF) [52,55]. The highest number of meningitis cases was reported during the 1980 outbreak in Pará, with 2% laboratory-confirmed cases (22 out of the 292 OROV-positive cases) [53]. Symptom recurrence is common, often occurring about 2–10 days after the acute phase [10]. However, attempts to isolate the virus during this period have been unsuccessful (personal communication, Instituto Evandro Chagas; Belém, Brazil), leaving the cause of recurrence unclear. This symptom recurrence has also been observed in 3 of the 32 OROV-positive US travellers [46]. Both men and women are equally affected, with those aged 20–49 years most susceptible, likely due to greater exposure to the vector [6,7, 54](Fig. 5b).

Until now, Oropouche fever has been considered a mild, self-limiting disease. However, the recent mortalities seen with a 24- and 21-year-old female in Bahia suggest otherwise [3,56] (Fig. 4). Both patients experienced fever, headache, retro-orbital pain, myalgia, severe abdominal pain, diarrhoea, nausea and vomiting. The second patient also presented with joint pain, a reddish rash, purple spots and bleeding from the nose, gums and vagina. Clinical reports note that both patients died 4 days after symptom onset. The first patient developed symptoms on 23 March 2024 and died on 27 March 2024, while the second patient died on 9 June 2024, after symptom onset on 5 June 2024. Both patients had high viral loads with Ct values of 16 and 8, respectively (Ct, cycle threshold for RT-qPCR assay). Both patients also suffered from rapid disease progression, severe coagulopathy, and liver impairment. No neurological symptoms were observed in either case, and neither had underlying health conditions [56].

In July 2024, two cases of suspected vertical transmission of OROV were reported in Pernambuco, Brazil [2] (Fig. 4). The first case involved a woman who experienced symptoms on 24 May 2024, leading to foetal death on 6 June 2024 at 30 weeks of gestation. vRNA was detected in the umbilical cord blood and various foetal tissues, including the foetal brain, liver, kidneys, lungs, heart and spleen. This is now the first confirmed case of OROV vertical transmission. The second case involved a woman who experienced symptom onset on 6 June 2024, followed by a miscarriage on 27 June 2024, at 8 weeks of gestation. Since then, two additional stillbirths have been reported. A retrospective study by the Instituto Evandro Chagas has now identified OROV IgM antibodies in serum (two cases) and the CSF (two cases) in three 1-day and one 27-day-old newborn with microcephaly. These samples had previously tested negative for dengue, chikungunya, Zika and West Nile viruses. At this time, aside from the initial confirmed case, all other instances of vertical transmission are still under investigation [2].

The clinical reports of the two mortalities note how the patient’s symptoms resembled severe dengue fever, marked by shock, bleeding and extensive coagulopathy, and suggest if not for the ongoing OROV outbreak, they most likely would have been misclassified as dengue [56]. RT-qPCR did not indicate co-infection with other arboviruses. OROV is often misclassified in regions with prevalent dengue, Zika, chikungunya and other arboviruses, thus raising the possibility that similar cases may have gone unrecorded in the past. Concerningly, haemorrhagic symptoms have previously been reported in Amazonas in 2007–2008 [17]. Symptoms in these patients appeared as petechiae, epistaxis and bleeding gums. Again in 2010 in Peru, cases of epistaxis, gingival and vaginal bleeding were observed [57]. No deaths were recorded in either instance. Potential cases of OROV vertical transmission may have also occurred in the past based on reports of the 1981 OROV outbreak, where spontaneous abortion was noted in two women who tested positive for the virus [58,59]. Vertical transmission of OROV would not be surprising, considering that closely related orthobunyaviruses affecting ruminants, such as the Schmallenberg virus, are known for vertical transmission [60]. Additionally, the phlebovirus Rift Valley fever virus is already established as being vertically transmitted in humans [61,63].

From personal communication in 2013 from the Department of Arbovirology and Hemorrhagic Fevers, Instituto Evandro Chagas, the routine diagnostic tests for febrile patients in northern Brazil include a haemagglutination inhibition (HI), complement fixation and enzyme-linked immunosorbent assay (ELISA). The HI assay screens for 19 different arboviruses but has low specificity, often resulting in cross-reactions. Viral isolation is attempted on OROV-positive samples collected within a 5-day window. Molecular diagnostics such as RT-qPCR assays are probably more common now and can screen for multiple arboviruses [64,65]. Reports of OROV being detectable in saliva and urine up to 5 days after symptom onset may also allow for alternative ways of OROV screening [66]. No antivirals or vaccines are available for OROV, and clinical management is limited to supportive care. Recent guidance (August 2024) from the Centers for Disease Control and Prevention advises OROV-positive individuals against non-steroidal anti-inflammatory drugs to reduce the risk of bleeding and recommends that pregnant individuals reconsider non-essential travel to Brazil and Cuba [46,67, 68].

## Biology and genetics of OROV

**Fig. 6.**
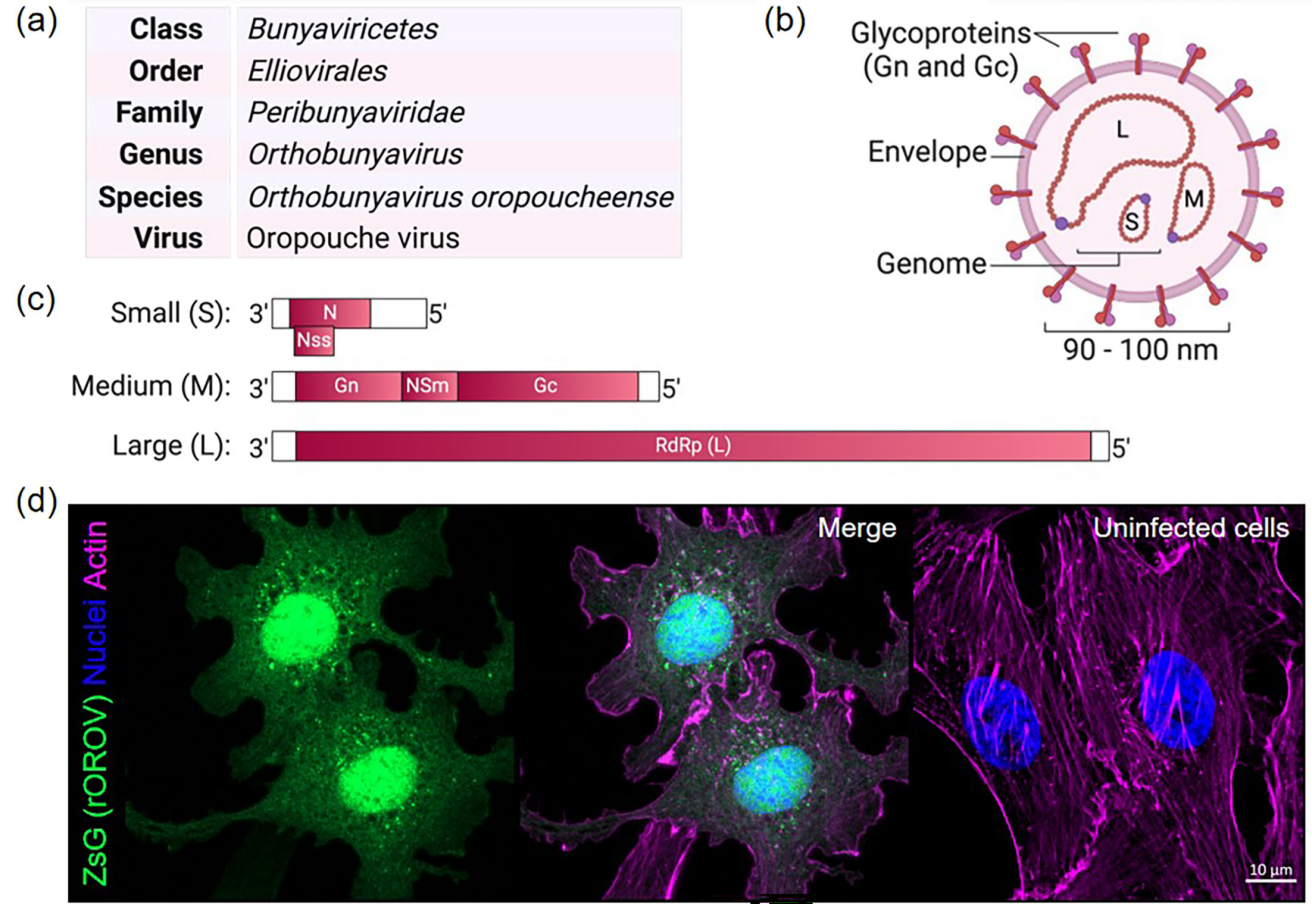
OROV biology. (**a**) Taxonomical classification of OROV. (**b**) OROV virion. The genome consists of S, small; M, medium and L, large segments. (**c**) Schematic of OROV genome. S encodes the nucleoprotein (n) and a non-structural (NSs) protein in overlapping reading frames. M encodes Gn, a non-structural protein (NSm), and Gc as a polyprotein, and L encodes the RNA-dependent RNA polymerase (RdRp, L protein). Untranslated regions (white boxes) flank the coding sequences. (**d**) Recombinant reporter OROV. Images collected from fluorescent reporter OROV-infected Vero-E6 cells 24 h post-infection [78]. Magnification 63X. (**a–c**) Created with Biorender.com.

OROV is a tri-segmented, single-stranded, negative-sense RNA virus within the *Peribunyaviridae* family and *Orthobunyavirus* genus [1] (Fig. 6a–c). Its genome and life cycle are conserved with other orthobunyaviruses, with few exceptions. The virus’s spherical, enveloped virions measure approximately 90–100 nm in diameter. Its genome consists of three segments: small (S; 958 nt), medium (M; 4385 nt) and large (L; 6852 nt). The S-segment encodes the nucleoprotein (N; ~27 kDa) and a non-structural protein (NSs; ~11 kDa) via overlapping open reading frames. The M segment encodes a glycoprotein precursor, which is co-translationally cleaved into glycoproteins Gn (~32 kDa) and Gc (~110 kDa), along with a non-structural protein (NSm; ~27 kDa). The L segment encodes the RNA-dependent RNA polymerase (RdRp; ~261 kDa). Untranslated regions (UTRs) flank the coding regions and contain signals essential for transcription, replication and packaging [69,70]. As a negative-sense virus, the OROV genome cannot directly serve as a transcriptional template. Instead, the N, the vRNA segments and RdRp assemble to form ribonucleoprotein complexes. These viral protein–RNA complexes facilitate genome replication and transcription in the cytoplasm of infected cells. Translation occurs via a cap-snatching mechanism in which short-capped primers from host mRNAs are cleaved by the RdRp. Viral mRNA translation then proceeds using the host’s protein synthesis machinery [70]. The resulting viral proteins and segments are trafficked to the Golgi apparatus for assembly and budding [70,72].

Our understanding of OROV biology is still limited, but we do know that the virus primarily enters host cells through clathrin-coated vesicles [73], utilizing the host protein low-density lipoprotein-related protein 1 (Lrp1) for efficient infection [74]. Despite low levels, OROV can infect T cells, monocytes, dendritic cells and B cells. This has led to the hypothesis that leukocytes may act as a Trojan horse, facilitating OROV dissemination within the host, particularly during immunosuppression [75]. In the brain, OROV predominantly targets microglia, highlighting its potential neurotropism [76]. Our work in developing the first OROV reverse genetics system [69,77] using the prototype strain BeAn19991 (Accession numbers: KP052850–KP052852) enabled us to demonstrate that the viral NSs protein is an interferon (IFN) antagonist, consistent with other NSs-encoding orthobunyaviruses. We identified a conserved motif at the NSs carboxy terminus critical for this IFN antagonistic activity [77]. We also found that the NSm protein is dispensable for OROV replication in mammalian cells [77]. Leveraging our NSm finding, we have now developed the first reporter OROV by substituting NSm with the green fluorescent protein ZsGreen [78]. This reporter OROV is an invaluable tool for pathogenesis studies and will help advance our understanding of OROV biology (Fig. 6d). Additionally, it will facilitate drug screening and neutralization assays, making it especially relevant in the context of the current outbreak.

## OROV evolution

**Fig. 7.**
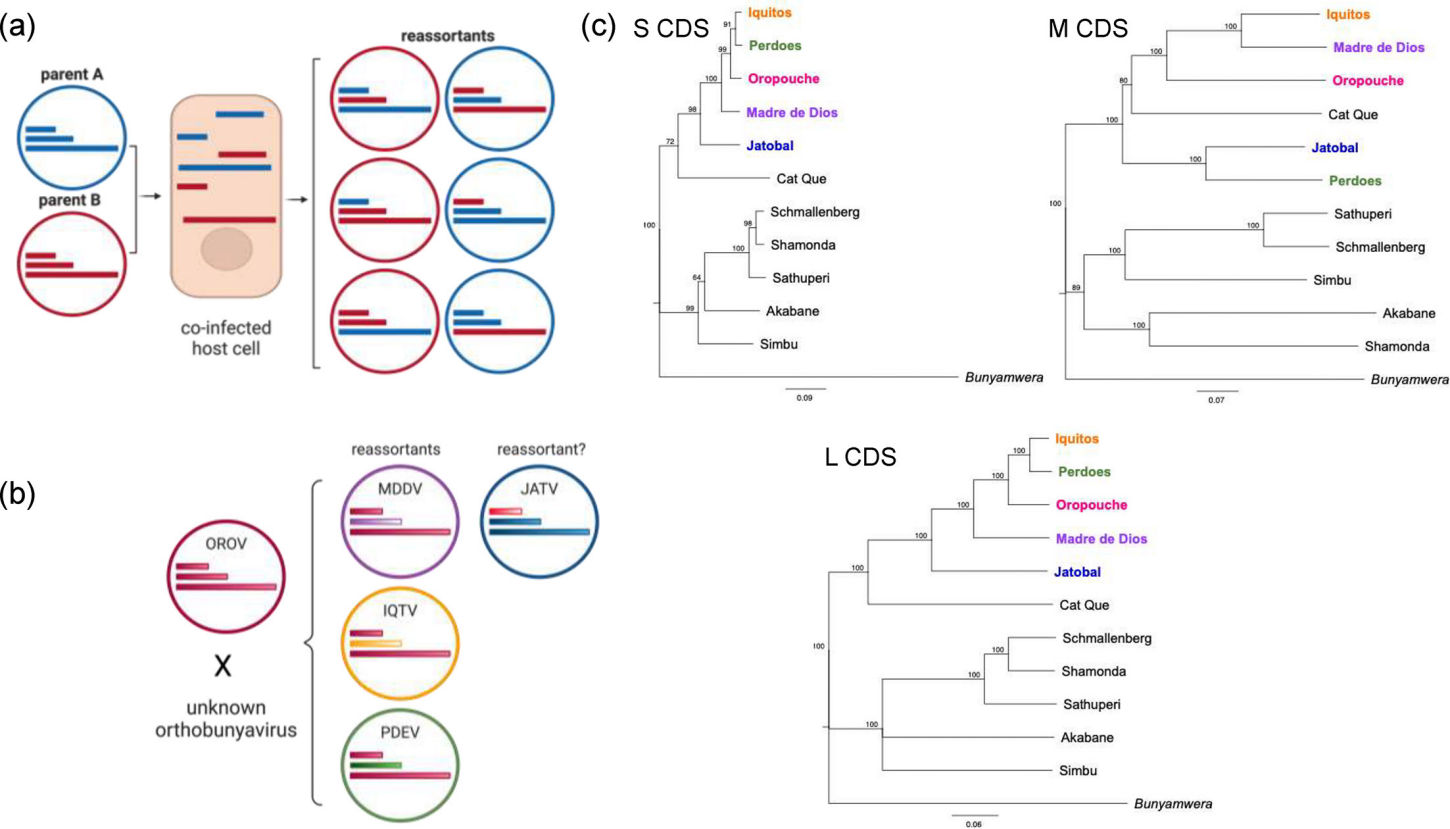
OROV reassortants. (**a**) Schematic representation of orthobunyavirus reassortment. (**b**) Schematic representation of OROV reassortants. OROV, Oropouche virus (pink); MDDV, Madre de Dios virus (purple); IQTV, Iquitos (yellow); PDEV, Perdões virus (green) and JATV, Jatobal virus (blue). (a) and (b) were created with Biorender.com. (**c**) Phylogeny depicting the evolutionary relationship of OROV, MDDV, IQTV, PDEV and JATV based on the S, M and L coding sequences (CDS). Trees were created using a maximum-likelihood method based on the general time reversible model in Geneious. Accession numbers: Iquitos, *S: KF697144, M: KF697143, L: KF697142*; Perdões, *S: KP691626, M: KP691625, L: KP691627*; Oropouche, S*: KP026181, M: KP026180, L: KP026179*; Madre de Dios, *S: KF697146, M: KF697145, L: KJ866391*; Jatobal, *S: JQ675601, M: JQ675602, L: JQ675603*; Cat Que, *S: OP129710, M: OP129711, L: OP129710*; Schmallenberg, *S: NC_043582, M: NC_043584, L: NC_043583*; Shamonda, *S: LC741389, M: LC741388, L: LC741387*; Sathuperi*, S: LC741386, M: LC741385, L: LC741384*; Akabane, *S: KY385908, M: KY381277, L: KY381282*; Simbu, S*: AF362397, M: NC_018478, L: NC_018476*; Bunyamwera, *S: NC_001927, M: NC_001926, L: NC_001925*.

The segmented nature of OROV’s genome allows for reassortment during co-infection. Here, co-infecting segmented viruses exchange genome segments, giving rise to novel gene combinations [79] (Fig. 7a). This genetic exchange can lead to significant evolutionary shifts and is a major driver of segmented virus evolution. While reassortment’s role in influenza virus host range and virulence is well documented [80], its impact on orthobunyaviruses remains poorly understood. For instance, several OROV reassortants have emerged outside traditional endemic areas over the past decade. Iquitos virus (IQTV, *O. oropoucheense*) and Madre de Dios virus (MDDV, *O. oropoucheense*) were isolated from people during OROV outbreaks in Peru in 1999 [81] and 2007 [82], respectively. MDDV was also isolated from a moribund capuchin monkey (*C. olivaceus*) during a fatal epizootic in Venezuela in 2010 [83]. In 2012, we discovered Perdões virus (PDEV, *O. oropoucheense*) in marmosets (*C. penicillate*) from Minas Gerais, Brazil [34]. MDDV, IQTV and PDEV share OROV’s S and L segments, while their M segments originate from unidentified orthobunyaviruses [34]. This is concerning given that the M segment encodes glycoproteins Gn and Gc, which are essential for viral attachment and entry into host cells [70]. Novel M segments could potentially alter OROV pathogenesis and/or vector competency, enabling OROV to emerge in new ecological niches. It is unclear whether Jatobal virus (JATV*, O. jatobalense*), isolated from a ring-tailed coati (*Nasua nasua*) in Brazil in 1985, is an S-segment OROV reassortant [82]. It is possible that JATV may have originated as an OROV reassortant but has since diverged further from OROV (Fig. 7b, c).

Inter-species reassortment events, such as those that produced MDDV, IQTV and PDEV, are rare but can drive significant evolutionary leaps, including cross-species transmission [79]. In contrast, intra-species reassortment occurs more frequently. Phylogenetic studies reveal distinct evolutionary trajectories for each OROV segment [34,84], with the M and L segments forming two major lineages and the S-segment forming three main lineages [18,34]. The current OROV outbreak likely resulted from complex reassortment events amongst these diverse OROV S, M and L lineages [18]. While there is no consensus on the classification of OROV lineages, the most recent comprehensive study by Naveca *et al*. [18] provides valuable insights into the complexity of OROV reassortment in South America. The study sequenced 382 clinical isolates across three Brazilian states between August 2022 and February 2024 and compared these with the 72 OROV sequences already in GenBank (NCBI). The 72 sequences encompass OROV isolates from 1955 to 2021. The findings suggest that the currently circulating isolates contain an M segment closely related to the most prevalent lineage in Brazil (the authors call this lineage 1). However, the S and L segments branch more closely with the 2008 and 2021 sequences isolated from Peru, Columbia and Ecuador (the authors call this lineage 2). It is important to note that the isolates circulating in those regions at the time were themselves probably reassortants. For a detailed account of these sequences and the proposed reassortment events, refer to the study by Naveca *et al*. [18].

Although intra-species reassortment events do not always lead to drastic phenotypic changes, the current reassortants show significant differences. For instance, serum from individuals infected during a 2016 OROV outbreak has shown a marked decrease in neutralizing ability against a current 2024 isolate (AM0088) compared to the prototype strain BeAn19991 [37]. An alignment of the M polyproteins reveals approximately 24 amino acid differences (across Gn, NSm and Gc) between AM0088 and BeAn19991, which could contribute to this reduced neutralizing ability. Understanding these contemporary isolates' pathogenicity and host and vector tropism is now a critical research imperative.

## Pathogenesis and transmission models

Animal models are crucial for studying OROV pathogenesis and evaluating new vaccines and therapeutics. Mice and hamsters are commonly used despite their limitations, as they are not naturally susceptible to OROV [5,85,89]. Adult immunocompetent BALB/c and C57BL/6 mice do not exhibit signs of illness but generate neutralizing antibodies, making them potentially useful for investigating the B- and T-cell responses, for instance. In contrast, subcutaneous inoculation of adult Syrian golden hamsters (*Mesocricetus auratus*) and IFN-α/β receptor knock-out mice (IFNAR^-/-^) leads to systemic infection, with symptoms appearing 72 h post-infection, followed by hepatitis and CNS damage. Neonatal BALB/c mice have been used to demonstrate OROV neurotropism. When these mice were inoculated with 10^6^ TCID_50_ of OROV, they succumbed within 10 days, demonstrating mild histopathological changes in the brain and spinal cord despite severe CNS disease [89]. Our recent work with our newly generated fluorescent OROV in IFNAR^-/-^ mice showed similar neurological outcomes, with mice primarily succumbing to severe liver necrosis between 5 and 8 days, depending on the viral dose [78]. Reporter viruses like ours enable real-time tracking and visualization of disease progression, suggesting that contemporary clinical isolate versions should be developed next. A list of animal models used for OROV research can be found in Table 2.

**Table 2.** Laboratory animal models of OROV disease

Animal	Route	Dose	Clinical signs	Mortality	Reference
Sucking Swiss mice	IC	Unclear	Irritability, prostration	Yes	[5]
Sucking Swiss mice	IP	Unclear	Irritability, prostration	Yes	[5]
Swiss mice	IC	Unclear	Irritability, prostration	Yes	[5]
Swiss mice	IP	Unclear	None	No	[5]
Guinea pigs	IC	5000 SM LD_50_	Hind limb paralysis	Yes	[5]
Guinea pigs	IP	50 000 SM LD_50_	None; neutralizing antibodies	No	[5]
Syrian golden hamsters	IP	Unclear	Hind limb paralysis, loss of appetite, ruffled fur, difficulty walking, prostration	2–4 days	[5]
Syrian golden hamsters	IC	Unclear	Hind limb paralysis, loss of appetite, ruffled fur, difficulty walking, prostration	2–4 days	[5]
Rabbits	IP	1000 SM LD_50_	None; neutralizing antibodies made	No	[5]
Rabbits	IC	80 000 SM LD_50_	None; neutralizing antibodies made	No	[5]
NHPs(Cebus monkeys)	IP	1000 SM LD_50_	None; virus detected in serum up to 5 days	No	[5]
Immunocompetent mice (C57BL/6)	SC (footpad)	10^6^ FFU	None	No	[85,86]
Immunodeficient mice (C57BL/6 IFNAR^-/-^)	SC (footpad)	10^6^ FFU	Weight loss	4–6 days	[85,86]
3-Week Syrian golden hamsters	SC	4 LD_50_(10^5.6^ TCID_50_)	Loss of appetite, ruffled fur, difficulty walking, high temperature, lethargy, shivering, ataxia, weight loss and hind limb paralysis	4–11 days	[87]
Immunodeficient mice (C57BL/6 IFNAR^-/-^)	SC	10 TCID_50_	Weight loss, ruffled fur, difficulty walking, lethargy and prostration	5–8 days	[78]
Immunodeficient mice (C57BL/6 IFNAR^-/-^)	SC	25 000 TCID_50_	Weight loss, ruffled fur, difficulty walking, lethargy and prostration	4–6 days	[78]

FFUfocus forming unitsICintracranialIPintraperitonealSCsubcutaneousSM LD50suckling mouse lethal dose 50TCID50tissue culture infectious dose 50

Transmission studies using animal [36] and human-to-animal models [90] in the 1980s confirmed that *Culicoides paraensis* can effectively transmit OROV. In the animal model, midges transmitted OROV 4–9 days after feeding on viraemic hamsters with viral titres between 6.7 and 9.9 log_10_ SM LD_50_ ml^−1^ (suckling mouse lethal dose). In the human-to-animal model, transmission to hamsters occurred at viral titres of 5.3 log_10_ SMLD_50_ ml^−1^, with successful transmission observed up to 6–12 days post-feeding. Laboratory experiments have shown that another midge, *Culicoides sonorensis*, is equally susceptible to OROV, with average viral titres reaching 2.5×10^4^ PFU ml^−1^ (plaque forming units). The virus disseminated well in these midges and was detectable in their legs and saliva. The competence of *Culicoides sonorensis* is particularly concerning due to its widespread range across North America. This species is the known vector for the closely related Schmallenberg virus in northern Europe [91].

Common laboratory mosquito species, such as *Aedes aegypti*, *Aedes albopictus*, *Culex quinquefasciatus* and *Culex tarsalis*, are mostly susceptible to OROV infection when directly injected rather than orally fed. However, when infected, they do not appear to be highly competent vectors, likely due to some midgut-level restriction [92,93]. This is interesting given that OROV-positive field-caught *Culex quinquefasciatus* mosquitoes have been found in Mato Grosso [94].

## Concluding remarks

OROV has posed a significant public health threat in Central and South America since the 1960s, yet the current outbreak has ignited interest within the research community. This outbreak underscores the urgent need for improved differential diagnostics, comprehensive epidemiological studies and genome sequencing to assess the true prevalence of OROV accurately. Critical questions in OROV biology remain unresolved, such as how the various glycoproteins encoded by different OROV reassortants influence tropism, pathogenesis, vector competency and cross-species transmission. A thorough biochemical assessment of viral and host protein interactions is necessary to elevate our current knowledge of OROV’s mode of action. Understanding the primary tissues infected by OROV, its neurotropic, hepatotropic and teratogenic potential, and the impact of NSs and NSm variants on viral–host interactions are crucial areas for further investigation. However, these studies require clinical isolates that have not been passaged in tissue culture. Much of the research conducted to date, including ours, has relied on the 1960 BeAn19991 isolate, a laboratory-adapted strain that complicates efforts to model the virus accurately. To develop effective vaccines and therapeutics, unpassaged clinical isolates that can be used to develop well-characterized recombinant viruses, and robust animal models are essential.

The upsurge in OROV cases may be linked to the heavier-than-usual rainfall from the 2023–2024 El Niño. However, we must anticipate OROV spreading beyond its traditional endemic regions due to anthropogenic ecological changes and global climate shifts [95]. A One Health approach is vital to preventing OROV outbreaks. This requires a thorough investigation into the various vectors and animal reservoirs of OROV [96]. This comprehensive understanding is essential to avoid OROV from becoming endemic outside South America.

## Abbreviations

CNS: central nervous system
CSF: cerebrospinal fluid
IEC: Instituto Evandro Chagas
IFN: interferon
IQTV: Iquitos virus
JATV: Jatobal virus
MDDV: Madre de Dios virus
mRNA: messenger ribonucleic acid
NSm: non-structural medium protein
NSs: non-structural medium protein
ORF: open reading frame
OROV: Oropouche virus
PAHO: Pan American Health Organization
PDEV: Perdoes virus
RdRp: RNA-dependent RNA polymerase
RT-qPCR: reverse transcription-quantitative polymerase chain reaction
vRNA: viral RNA
WHO: World Health Organization
